# Comparison of Prehospital Versus Arrival Glasgow Coma Scale Scores Among Trauma Patients: A Retrospective Analysis

**DOI:** 10.7759/cureus.92364

**Published:** 2025-09-15

**Authors:** Timothy M Attride, Ryan M Hatfield, Nathan A Huffman, Deborah M Hurley, Spencer F Robinson

**Affiliations:** 1 Department of Emergency Medicine, Prisma Health Midlands, Columbia, USA; 2 Post-baccalaureate Premedical Program, University of Michigan, Ann Arbor, USA; 3 Department of Research Services, Prisma Health Midlands, Columbia, USA

**Keywords:** emergency medical services, ems, gcs, glasgow coma scale, trauma

## Abstract

Introduction: The Glasgow Coma Scale (GCS) is a valuable tool that has long been used to help identify critically ill trauma patients in the prehospital setting. A discrepancy was observed by emergency medicine staff in prehospital GCS (pGCS) versus arrival GCS (aGCS). This observed discrepancy in GCS has important implications in the proper triage of trauma patients, including the appropriate level of activation, resource utilization, and cost.

Study objective: The aim of the study was to determine the level of agreement between pGCS and aGCS among trauma patients, determine the effect of alcohol intoxication on GCS agreement, analyze for differences in GCS among methods of transport, and limit confounding variables in the patient population.

Methods: A single-center retrospective cohort of 2,531 trauma patients aged 14 and older who presented as level 1 and level 2 trauma alerts was included. To determine agreement, component, total, and categorical GCS scores were compared using weighted Cohen’s kappa. In addition, the effect on agreement among acutely intoxicated trauma patients was assessed. Finally, absolute differences in mean scores stratified by method of transport were also evaluated.

Results: The study included 2,531 patients. Overall agreement between pGCS and aGCS was moderate (kappa 0.54) with substantial agreement (kappa 0.67) when categorized by severity. Alcohol intoxication decreased agreement to moderate agreement (kappa 0.57). pGCS tended to be lower than aGCS for ground Emergency Medical Services (EMS) (-0.45) and higher for air EMS (0.7).

Conclusion: GCS agreement between prehospital and emergency department (ED) providers is similar, with variation among methods of transport and patients who are acutely intoxicated; however, this difference may not be clinically significant.

## Introduction

The Glasgow Coma Scale (GCS) is a valuable tool for assessing neurologic status and overall clinical condition in the emergency department (ED). This tool has significant implications, including the decision for trauma team activation, the level of activation, and accurately conveying the clinical condition of the patient to the receiving trauma team. A GCS of <14 has been shown to predict the need for full trauma activation accurately and is the only reliable predictor of hospital admission in motor vehicle accidents [1]. Accurate usage of the scoring system is important given the considerable facility personnel and financial burden associated with trauma activations. A study assessing the costs of trauma center readiness in the United States found the average annual cost of level 1 trauma center readiness was approximately six to 10 million dollars per year [2]. A recent review of trauma activation fees found the average cost for a level 1 trauma activation was $9,500 per activation [3].

Discrepancies exist between the GCS reported by Emergency Medical Services (EMS) and the GCS score determined by ED personnel on arrival. Several potential explanations exist for the difference in reported GCS, including patient improvement following EMS intervention, patient decompensation during transport, level of clinical training, clinical experience of prehospital providers, and the inability to accurately calculate a GCS due to unfamiliarity with the scoring system and/or a need for additional GCS training. Refresher training has been shown to be effective in improving GCS calculation [4,5]. Previous research has shown that prehospital GCS cores (pGCS) are highly predictive of arrival GCS scores (aGCS), but this study asks, to what degree do pGCS and aGCS agree? Previous studies have shown pGCS is approximately two points lower than aGCS overall [6,7]. Another study found greater agreement between pGCS and aGCS with mild injury (GCS score of 13-15) when compared to moderate injury (GCS score of 9-12) and severe injury (GCS score of 3-8) [8].

The purpose of this study is to evaluate the agreement between GCS scores calculated by EMS (pGCS) and GCS scores calculated by providers on arrival to the hospital (aGCS). The study also aims to analyze the difference in GCS agreement between the air and ground EMS systems serving the study center, a level 1 trauma center, and to determine the effect of alcohol intoxication on GCS agreement.

## Materials and methods

Study design

This study is a single-center retrospective cohort study conducted at Prisma Health Midlands, a level 1 trauma center in Columbia, SC, USA, utilizing electronic health record reviews of patients presenting as trauma alerts from July 2019 to July 2023. These records were contained within the trauma center database. A total of 10,233 trauma patients were screened, and 2,531 patients met the inclusion criteria. Patients were eligible for the study if designated as either a level 1 or level 2 trauma alert and were aged 14 years or older. The study center resides in an urban area and is served by two air EMS systems and multiple ground EMS systems. Most trauma patients presenting by ground transport present by the county EMS system in which the study center resides. The method of transport was determined by the final transport method to the study center. Prehospital providers may not have the same level of confidence in GCS calculation in pediatric populations, and thus, the age of 14 was used as a cutoff. At the study center, level 1 is the highest activation level, and level 2 involves less severely injured patients. Both levels of alert trigger activation of the trauma team. Patients were excluded based on the following criteria: patients who suffered traumatic arrest en route, patients without a pGCS, patients without an aGCS, patients 13 and under, any patient pronounced dead in the trauma bay, and all trauma transfers from outside hospitals. Trauma transfers do not trigger the same alert in terms of resources required and associated cost; thus, this population was not included. Patients were screened for the inclusion and exclusion criteria using data collected from the trauma registry.

Ethical approval

The study received ethical approval from the institution’s IRB review committee without continuing review (approval number: 2078846-1). All trauma alerts generate an anonymous patient name and a standardized birthday, not specific to the patient. If the patient’s demographics are later known, the above outcome measures are collected, and all electronic demographic data are then de-identified.

Data collection

This study included GCS scores obtained by EMS prior to arrival and GCS scores documented by the trauma team on arrival for patients who met the above inclusion criteria. Only the initial calculated GCS in the prehospital system that was reported and triggered the trauma alert was used in this study; subsequent GCS calculations obtained prior to arrival were not included. Additional outcome measures were collected (Table 1) via retrospective chart review and review of the trauma registry database. Alcohol levels were measured upon arrival as a continuous variable via blood draw in the trauma bay and measured in mg/dL.

**Table 1:** Outcome Measures

Outcome Measures
Agency or Emergency Medical System (EMS) System
Mechanism
Prehospital Glasgow Coma Scale (pGCS)
Age
Respiratory Rate
Injury Severity Score (ISS)
Alcohol Level
Method (Ground vs. Air)
Injury
Arrival GCS (aGCS)
Blood Pressure
Oxygen Saturation
Date of Death

Statistical analysis

Agreement between pGCS and aGCS scores was evaluated using weighted Cohen’s kappa with 95% confidence intervals (CIs). Weighted Cohen's kappa corresponds to a level of agreement (Table 2). Individual component and total GCS scores were analyzed separately for absolute agreement. Further analyses were performed by EMS mode of transport (ground or air) and the effects of alcohol. Alcohol values are continuous values, and a cutoff of 80 mg/dL was used, as this is the legal cutoff for operating a vehicle in the study center state. Mean scores with 95% CIs were also used to compare differences in pGCS and aGCS for overall scoring and scoring stratified by method of transport. Data were analyzed in SAS software, version 9.4 (© 2019-2020, SAS Institute Inc., Cary, NC, USA).

**Table 2:** Weighted Cohen’s Kappa Value With Levels of Agreement

Weighted Cohen's Kappa Value	Level of Agreement
<0	None
0.01-0.20	None to Slight
0.21-.0.40	Fair
0.41-0.60	Moderate
0.61-0.80	Substantial
0.81-1.00	Almost Perfect

## Results

A total of 2,531 patients were identified as meeting the inclusion criteria. The average pGCS was 12.91 (±0.2), and the average aGCS was 13.2 (±0.15). Moderate agreement (0.47 to 0.54) was found among individual component and total GCS scores; however, agreement improved to substantial (0.67) when GCS was categorized by severity (mild, moderate, and severe) (Table 3). There was also higher absolute agreement with categorization (85.9%). Improvements in absolute agreement and kappa agreement with categorization declined significantly when acute alcohol intoxication was present, decreasing agreement from substantial to moderate (0.67 to 0.57) and decreasing absolute agreement (85.9% to 78.2%) (Table 4).

**Table 3:** Absolute Agreement and Weight Cohen’s Kappa Agreement GCS: Glasgow Coma Scale

Factor	N	%	Weighted Cohen's Kappa (95% CI)	Agreement
GCS Eye Agreement				
Yes	1868	79.5	0.48 (0.45, 0.51)	Moderate
No	483	20.5		
GCS Verbal Agreement				
Yes	1669	71	0.50 (0.48, 0.52)	Moderate
No	682	29		
GCS Motor Agreement				
Yes	1869	79.5	0.47 (0.45, 0.49)	Moderate
No	482	20.5		
GCS Total Agreement				
Yes	1474	62.7	0.54 (0.52, 0.55)	Moderate
No	877	37.3		
GCS Total Agreement (By Ground)				
Yes	1280	64	0.52 (0.51, 0.53)	Moderate
No	720	36		
GCS Total Agreement (By Helicopter)				
Yes	193	55.3	0.56 (0.54, 0.59)	Moderate
No	156	44.7		
GCS Total Agreement (Mild/Moderate/Severe)				
Yes	2019	85.9	0.67 (0.64, 0.69)	Substantial
No	332	14.1		

**Table 4:** GCS Total Agreement in Patients With Alcohol Levels Exceeding 80 mg/dL GCS: Glasgow Coma Scale

Factor	N	%	Weighted Cohen's Kappa (95% CI)	Agreement
GCS Total Agreement (Alcohol ≥80 mg/dL)				
Yes	262	45.7	0.48 (0.46, 0.50)	Moderate
No	311	54.3		
GCS Total Agreement (Mild/Moderate/Severe) (Alcohol ≥80 mg/dL)				
Yes	448	78.2	0.57 (0.53, 0.62)	Moderate
No	125	21.8		

GCS scores were also stratified by method of transport. Overall, pGCS scores were slightly lower than aGCS scores (-0.28 ±0.10); however, results differed by mode of transport. For ground EMS, results were similar to overall scores, with pGCS scores slightly lower than aGCS scores (-0.45 ±0.10). For air EMS, pGCS scores were slightly higher than aGCS scores (0.7 ±0.29) (Table 5).

**Table 5:** EMS GCS Mean Difference (95% CI) EMS: Emergency Medical Services; GCS: Glasgow Coma Scale

Transport Method	EMS GCS	ED GCS	Difference (EMS-ED)
Ground or Air			
Eye	3.48 (3.44, 3.52)	3.54 (3.51, 3.58)	-0.07 (-0.1, -0.04)
Verbal	4.14 (4.09, 4.2)	4.26 (4.2, 4.31)	-0.11 (-0.15, -0.07)
Motor	5.3 (5.24, 5.36)	5.4 (5.34, 5.46)	-0.11 (-0.15, -0.06)
Total	12.92 (12.77, 13.07)	13.2 (13.06, 13.35)	-0.28 (-0.39, -0.18)
Ground			
Eye	3.53 (3.49, 3.57)	3.63 (3.59, 3.67)	-0.1 (-0.13, -0.07)
Verbal	4.2 (4.15, 4.26)	4.37 (4.32, 4.42)	-0.17 (-0.21, -0.12)
Motor	5.35 (5.29, 5.42)	5.54 (5.49, 5.6)	-0.19 (-0.24, -0.14)
Total	13.09 (12.94, 13.24)	13.54 (13.4, 13.68)	-0.45 (-0.56, -0.35)
Air			
Eye	3.18 (3.05, 3.31)	3.04 (2.9, 3.18)	0.13 (0.05, 0.21)
Verbal	3.79 (3.63, 3.95)	3.59 (3.41, 3.78)	0.2 (0.08, 0.31)
Motor	4.98 (4.78, 5.17)	4.61 (4.38, 4.83)	0.37 (0.23, 0.51)
Total	11.94 (11.49, 12.4)	11.24 (10.71, 11.78)	0.7 (0.42, 0.99)

## Discussion

GCS is a proven predictor of mortality among traumatic brain injury (TBI) patients and has been proven to be the only reliable predictor of hospital admission after motor vehicle collision (MVC) [1,9]. This suggests GCS helps to predict more seriously injured patients. The patient’s injury, vital signs, and GCS have important implications for trauma alerts. Inaccurately low prehospital GCS scores can lead to overtriage, creating unnecessary costs and improper resource utilization for trauma patients. At the study trauma center, GCS <= 8 alone triggers a level 1 trauma activation. This retrospective analysis began with the presumption that pGCS reporting was inaccurate and led to inappropriate trauma activations, leading to unnecessary costs and overutilization of critical resources. However, this retrospective analysis showed higher levels of GCS agreement than expected, suggesting the founding hypothesis of this study was inaccurate.

A robust amount of literature exists regarding GCS and TBI with attempts to predict neurological outcomes [6]. One study in Australia analyzed prehospital vital signs and GCS scores in trauma patients. The authors found agreement but did not quantify the level of agreement [4]. Another study found that pre-hospital GCS scores were two points lower than those of emergency physicians; however, this study had a small sample size (60 patients) and only included patients with a GCS score of eight to 13 [7]. Another article published in 2006 demonstrated that the field GCS helps predict the aGCS, showing agreement with a strong correlation [6]. The Journal of Trauma conducted a similar study, using weighted Cohen’s kappa analysis for agreement, which yielded similar results to this study. However, the study had a different analytical design and did not analyze the effects of alcohol intoxication on agreement [7]. There remains a dearth of recent literature comparing GCS scoring among trauma patients in the United States, as most published studies are nearly or over 20 years old. There has also been a lack of focus on the effects of alcohol on GCS agreement, which is a common confounder within the trauma patient population. Among the study population, 24% had alcohol above the legal limit to drive, and this had a significant effect on GCS scoring, decreasing kappa agreement from substantial to moderate and absolute agreement by 10% (0.67 vs. 0.57). This significant disagreement certainly could lead to overtriage and inappropriate trauma activations.

The data showed much higher levels of agreement than expected, with substantial agreement and higher absolute agreement (85.9%) for categorical GCS scoring. This demonstrates that prehospital providers within this system are frequently able to accurately communicate the level of neurological injury for trauma patients, likely leading to appropriate levels of triage and trauma activations. pGCS was found to be slightly lower than aGCS overall (-0.28 ±0.10), but the average scores of ground EMS and air EMS differed. Ground EMS mean scores tended to be lower (-0.45 ±0.10) than air EMS scores (0.7 ±0.29). This difference was somewhat unexpected, and several theories have been proposed to explain it. Practically, air EMS was felt to be more liberal with the treatment of acute agitation with pain medication and rapid sequence intubation (RSI), given the inherent dangers of patient agitation within a helicopter. Flight crews typically have more latitude in patient care guidelines and less expectation for direct/online medical control for pain management or patient intervention. On average, flight crews (typically composed of a flight nurse and a flight paramedic) have greater experience and level of training compared to ground crews. These interventions and differing levels of experience could certainly lead to differences in agreement with lower aGCS; however, this difference warrants further research. Ground EMS pGCS closely reflected aGCS, which may be explained by shorter transport times, more restrictive patient care guidelines for treating acute pain, and a lower propensity for RSI in the field.

Naturally, patients will tend to experience a change in their GCS over time, depending on interventions or decompensation, and this likely contributes to a decrease in both absolute and weighted Cohen’s kappa agreement. The genesis of the study began with an anecdotal observation of poor overall agreement in GCS between the prehospital setting and arrival; however, the authors believe that the level of agreement is more than acceptable, given the limitations and confounding variables.

Limitations

This study has several limitations. Single-center study results may not be applicable to other systems with differing protocols and system design. The GCS score is a subjective rating that can vary marginally between providers. The level of training and experience with calculating GCS likely impacts scoring accuracy and precision; however, these were not controlled for. The high-stress environment and time constraint of performing multiple tasks simultaneously in prehospital care could lead to inaccurate depictions of certain GCS factors, which may skew specific data points. Transport times were also not controlled for, and longer transport times have the potential to decrease the level of GCS agreement due to patient decompensation or improvement based on provider intervention en route. Similarly, injury severity scores (ISS) were not controlled for, and those with higher ISS may tend to have lower levels of agreement. Certainly, EMS interventions such as intubation, medication administration, and fluid resuscitation can alter a patient's neurological status and decrease agreement with aGCS scores, and these were not controlled for.

Recreational drugs could also play a role in GCS agreement, and drug use is not an uncommon phenomenon among the trauma patient population. This variable is challenging to account for, as the most common testing is qualitative only and does not help determine acute intoxication.

In an effort to limit confounding variables, the authors excluded children, as many prehospital providers feel more comfortable with GCS calculation in adults, and differences in calculations would likely have been greater among the pediatric population. The authors also tried to further limit confounding variables by excluding patients who would have a significant or rapid change in GCS: patients who suffered traumatic arrest en route and those declared dead on arrival. Excluding these populations could introduce bias and artificially increase levels of agreement in the study. Lastly, previous medical history is difficult to control for, and pre-existing conditions, including previous stroke, psychiatric disorders, concomitant medication overdose, or other acute medical emergencies, could lead to decreased GCS agreement.

## Conclusions

This study began with the hypothesis that there was poor overall agreement between pGCS and aGCS within the single-center study system; however, our study found categorical GCS to be substantial in non-intoxicated trauma patients, with high levels of absolute agreement. Alcohol had a significant effect, decreasing agreement to only moderate, which could certainly lead to overtriage and inappropriate trauma activations. Air and ground EMS had similar overall agreement; however, they had differing absolute pGCS scores, with ground EMS scores closely reflecting aGCS, and air EMS scores tending to be higher than aGCS. This may be attributed to greater ability for intervention en route compared to ground EMS, and this area would benefit from future research. These results may differ in systems with alternative structures, designs, and differing transport times, which limits general applicability to other trauma care systems. Overall, the authors believe that the GCS agreement was more accurate than expected, resulting in frequent appropriate levels of triage and trauma activations.
